# Efficacy of Emergency Bypass System on hemodynamic instability during off-pump coronary artery bypass: propensity matched analysis

**DOI:** 10.1186/1749-8090-10-S1-A39

**Published:** 2015-12-16

**Authors:** Cheong Lim, Hyun Oh Park, Jung hee Kim, Sue Hyun Kim, Su Ryeun Chung, Dong Jung Kim, Jun Sung Kim, Kay-Hyun Park, Yong Jin Kim

**Affiliations:** 1 Department of Thoracic and Cardiovascular Surgery, Seoul National University Bundang Hospital, South Korea

## Background/Introduction

Hemodynamic instability during off-pump coronary artery bypass (OPCAB) is usually solved by intra-aortic balloon pump or on-pump conversion.

## Aims/Objectives

We tried to evaluate the usefulness of Emergency Bypass System (EBS)-supported beating heart coronary artery bypass (EBS-CAB).

## Method

We retrospectively reviewed medical records of 398 consecutive patients who underwent isolated elective OPCAB with intention-to-treat base from July 2005 to March 2015. 365 patients underwent successful OPCAB, whereas 24 patients were converted to EBS-CAB due to hemodynamic instability. The early and late clinical outcomes were analyzed and then propensity matched analysis was performed to minimize selection bias (39 patients from OPCAB group versus 20 patients from EBS-CAB group).

## Results

Mean age was 65.8 ± 9.7 years in OPCAB group and 71.7 ± 7.1 years in EBS-CAB group (p = 0.001). Preoperatively, incidence of hypertension, diabetes, stroke, renal failure, left ventricular dysfunction, and myocardial infarction was similar in two groups. However, Euroscore was significantly lower in OPCAB group (3.2 ± 2.4 vs 5.1 ± 2.4, p = 0.001). Postoperatively, both groups showed similar incidence of complications such as stroke, atrial fibrillation, mediastinitis and bleeding. Operative mortality and survival rate was not different on Kaplan-Meier analysis (Figure 1). Also, propensity-matched analysis showed similar results.

## Discussion/Conclusion

Patients who underwent EBS-CAB had similar postoperative outcomes compared with OPCAB despite of intraoperative hemodynamic derangement. EBS-CAB can be valuable option to minimize the operative risk when patients had unstable vital sign during OPCAB.

